# Simultaneous Reconstruction of Multiple Time-Varying Thermal Properties Based on Translucent Materials

**DOI:** 10.3390/ma17092088

**Published:** 2024-04-29

**Authors:** Fangxu Dong, Limei Fan, Jian Duan, Fei Wang, Junyan Liu, Yan Sun, Zhenhe Tang, Liangwen Sun

**Affiliations:** 1 Shandong Nonmetallic Materials Institute, Jinan 250031, China; limei8729@126.com (L.F.); duanjian_@163.com (J.D.); wosyh@163.com (Y.S.); jinan53tang@126.com (Z.T.); slw53nlyz@163.com (L.S.); 2 School of Mechatronics Engineering, Harbin Institute of Technology, Harbin 150001, China; wangfeipublic@163.com (F.W.); ljywlj@hit.edu.cn (J.L.)

**Keywords:** participatory medium, radiation–conduction coupling, inverse problem, multi-parameter reconstruction of time-varying photothermal physical properties

## Abstract

In the realm of high-tech materials and energy applications, accurately measuring the transient heat flow at media boundaries and the internal thermal conductivity of materials in harsh heat exchange environments poses a significant challenge when using conventional direct measurement methods. Consequently, the study of photothermal parameter reconstruction in translucent media, which relies on indirect measurement techniques, has crucial practical value. Current research on reconstructing photothermal properties within participating media typically focuses on single-objective or time-invariant properties. There is a pressing need to develop effective methods for the simultaneous reconstruction of time-varying thermal flow fields and internal thermal conductivity at the boundaries of participating media. This paper introduces a computational model based on the numerical simulation theory of internal heat transfer systems in participating media, stochastic particle swarm optimization algorithms, and Kalman filter technology. The model aims to enable the simultaneous reconstruction of various thermal parameters within the target medium. Our results demonstrate that under varying levels of measurement noise, the inversion results for different target parameters exhibit slight oscillations around the true values, leading to a reduction in reconstruction accuracy. However, overall, the model demonstrates robustness and accuracy in ideal conditions, validating its effectiveness.

## 1. Introduction

Translucent media, positioned between transparent and opaque media, are characterized by limited optical thickness within specific wavebands. These materials are also known as particle-containing, participating, absorbing and scattering, or dispersing media, depending on the research domain or target [1]. Examples of translucent media range from common substances like water, glass, air, and biological tissues to specialized materials used in aerospace, defense, and military applications. In these sectors, translucent materials play vital roles in various components such as spacecraft thermal protection materials, rocket engine plumes, and ceramic parts for high-temperature environments [2,3,4,5,6,7,8]. Research on the photothermal parameter field reconstruction of translucent media is significantly valuable for advanced technology, non-destructive testing, medical imaging, and clinical practices. For instance, the development of efficient thermal protection materials is essential for spacecraft re-entry scenarios in which extreme aerodynamic heating occurs. Understanding photothermal properties such as the thermal conductivity, absorption coefficient, and temperature distribution is crucial to ensuring the suitability of materials for withstanding high-temperature environments and designing effective thermal protection systems for aerospace applications [9,10,11,12,13]. Accurate measurements of these physical parameters are essential for evaluating the thermal performance of translucent materials and optimizing their use in real-world applications.

The increasing complexity of heat transfer systems across various fields has posed challenges in exploring the thermophysical properties of materials at high temperatures using traditional direct contact measurement methods. As a result, researchers worldwide have focused on employing the concept of inverse heat transfer problems to deduce the thermal properties of materials. The inverse heat transfer problem involves utilizing observable information from a system to reconstruct and calculate internal characteristics or boundary parameters. The solution is often unstable and non-unique compared to the forward heat transfer problem. Cui et al. [14] solved the heat conduction equation of the second type of boundary condition based on the implicit finite difference method and used the quantum behavioral particle swarm algorithm to invert the material physical properties with a high level of accuracy. Zhou et al. [15] solved the problem of non-unique solutions when converting thermophysical parameters from the space domain to the temperature domain by adding the constraint that the thermophysical parameters are piecewise functions of the temperature. Yin et al. [16] used Abaqus 2019 to solve the forward problem based on a heat-proof tile structure, combined with Isight 2019 for the inversion, and used the LM algorithm to effectively invert the identification parameters. Tahmasbi et al. [17] proposed using the idea of the inverse problem of heat conduction to invert the thermophysical parameters of carbonized composite materials. Considering that carbonized composite materials are orthotropic materials, they established a simultaneous inversion of the thermal conductivity coefficient in the plane and thickness directions using the multidimensional inversion method. Yan et al. [18] used the cuckoo algorithm as the optimization algorithm to establish a mathematical model for the nonlinear two-dimensional steady-state heat conduction inverse problem and discussed the impact of the number of units, the number of measuring points, the number of nests, and the measurement error on the inversion results. Yang et al. [19] proposed an improved conjugate gradient algorithm to identify the thermal physical property parameters of transient heat conduction problems when dealing with thermal conduction problems in engineering composite materials. In the forward problem, the integral method is used to obtain the temperature field of transient heat conduction based on the radial boundary element method. In the inverse problem, the complex variable function is introduced into the traditional conjugate gradient method, which improves the accuracy when calculating the sensitivity matrix. Niu et al. [20] proposed a method that combines the Gaussian parameter level set method and regularized Landweber algorithm to reconstruct the temperature and concentration distribution of the turbulent reaction flow, which makes up for the lack of prior knowledge of the inverse problem of tomography. Lou et al. [21] studied the inverse problem of coupled heat transfer in one-dimensional emission, absorption, and isotropic scattering participating media. The scattering coefficient and absorption coefficient of the media were inverted by the decoupling reconstruction algorithm, and the temperature field inside the media was reconstructed by the Tikhonov regularization method. Cui et al. [22] solved the forward problem of radiative–thermal coupling heat transfer by using the domain method coupled with the finite difference method.

In summary, current research on identifying the thermal physical property parameters of materials primarily focuses on a single-objective parameter inversion, with limited studies focusing on reconstructing photothermal parameters that vary over time. The simultaneous reconstruction of multiple targets within translucent media holds significant theoretical importance for applications such as the non-destructive testing of translucent materials, high-temperature flame combustion diagnosis, and biological tissue tumor diagnosis and therapy. There is a need for in-depth research on time-varying multi-parameter field reconstruction in translucent media to enhance its practical application value. This paper conducts a simultaneous optimization and inversion study on the time-varying boundary heat flow and various internal thermal characteristics of the target translucent material and finally verifies the accuracy and robustness of the established model.

## 2. Materials and Methods

### 2.1. Forward Model

**Figure 1:**
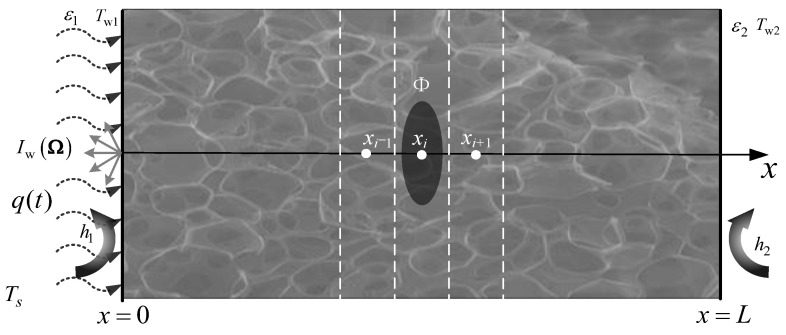
One-dimensional translucent medium heat conduction system.

Given an infinite plate of translucent material, the temperature gradient in the x-direction significantly exceeds that in the other two directions. Consequently, the heat transfer system can be simplified to a one-dimensional heat conduction problem, as illustrated in Figure 1. The diagram depicts a time-varying high heat flux applied to the left boundary of the medium. Natural convection heat transfer takes place between the medium boundary and the environment, with a constant heat transfer coefficient. Furthermore, to simplify the mathematical analysis, the following assumptions are employed: (1) the physical properties of the medium are constant, implying that its dimensions remain unchanged with temperature variations; (2) there is no phase change within the medium throughout the heat transfer process; and (3) the medium boundary is considered an opaque gray body boundary.

The energy equation of this model in Cartesian coordinate system can be expressed as follows:

(1) $$\rho c_{p}\frac{\partial T\left(x,t\right)}{\partial t}=\frac{\partial }{\partial x}\left[\lambda \frac{\partial T\left(x,t\right)}{\partial x}\right]-\frac{\partial q^{r}}{\partial x}+\Phi$$

where *ρ* represents the density of the medium, kg/m^3^; *c*_p_ represents the specific heat capacity of the medium, J/(kg·K); *T*(*x*,*t*) represents the temperature of point *x* of the medium at time *t*, K; *λ* represents heat conduction coefficient, W/(m·K); *q^r^* represents the radiant heat flow; and Φ indicates the internal heat source.

For opaque diffuse boundaries, the boundary conditions of the energy equation can generally be expressed as follows:

(2) $$\varepsilon \sigma \left(T_{s}^{4}-T^{4}\right)+\varepsilon_{w}\left(q^{r}-\sigma T^{4}\right)+q(t)+q^{\mathrm{cv}}+q^{\mathrm{cd}}=0$$

where *q*(*t*) represents the heat flow on the surface of the medium and *ε*_w_ represents the wall emissivity. *T*_w_ and *T_s_* refer to medium boundary temperature and ambient temperature, respectively. *q*^r^, *q*^cv^, and *q*^cd^ refer to radiative heat transfer, convective heat transfer, and thermal heat flow on the surface of the medium, respectively.

The corresponding initial conditions can be expressed as follows:

(3) $${\left.T\left(x,t\right)\right|}_{t=0}=T_{0}$$

The corresponding boundary conditions can be expressed as follows:

(4) $$-\lambda \frac{\partial T\left(x,t\right)}{\partial x}=q\left(t\right)-h_{1}(T_{e1}-T_{w1})-\varepsilon_{1}\sigma \left(T_{e}^{4}-T_{w}^{4}\right)\;\;\;\;\;\;\;\;\;\;\;\;\;\;x=0$$

(5) $$-\lambda \frac{\partial T\left(x,t\right)}{\partial x}=-h_{2}\left(T_{e2}-T_{w2}\right)+\varepsilon_{2}\sigma \left(T_{w}^{4}-T_{e}^{4}\right)\;\;\;\;\;\;\;\;\;\;\;\;\;\;x=L$$

where *q*(*r*,*t*) represents the transient heat flow acting on the left boundary, W/m^2^; *h* represents the convection heat transfer coefficient; and *T_e_* and *T_w_* represent the ambient temperature and wall temperature, respectively, K.

The inner node method is used to discretize the solution region, and the radiative transfer equation and the energy equation are calculated by the discrete coordinate method and the finite volume method, respectively, to obtain the temperature field of the medium.

### 2.2. Inversion Model

Define the objective function as follows:

(6) $$F(x)=\frac{1}{2}{\left[\frac{M-P\left(x\right)}{M}\right]}^{2}$$

where *x* is the characteristic parameter to be inverted, *M* represents the measured signal, and *P* indicates the predicted signal during the inversion calculation process.

If the predicted target parameters fall within the predetermined error threshold, they are considered close to the actual target characteristics. Otherwise, an iterative correction of the parameter prediction values is necessary. The predictive distribution of characteristics undergoes repeated iterative optimization until final target functional size meets the specified threshold, signifying the completion of thermal characteristics′ inversion calculation.

#### 2.2.1. SPSO Model

If Newton’s iteration method or its improved version is used, the results obtained are often locally optimal because they depend on the selection of initial parameters. Therefore, once there is a problem with the selection of parameters, the solution will fail. The particle swarm algorithm (PSO) [23] is an excellent intelligent computing technology, which is characterized by small population size, low initial parameter requirements, stable operation, simple structure, fast convergence, and easy operation. Therefore, it has great practical significance when dealing with complex nonlinear problems.

**Figure 2:**
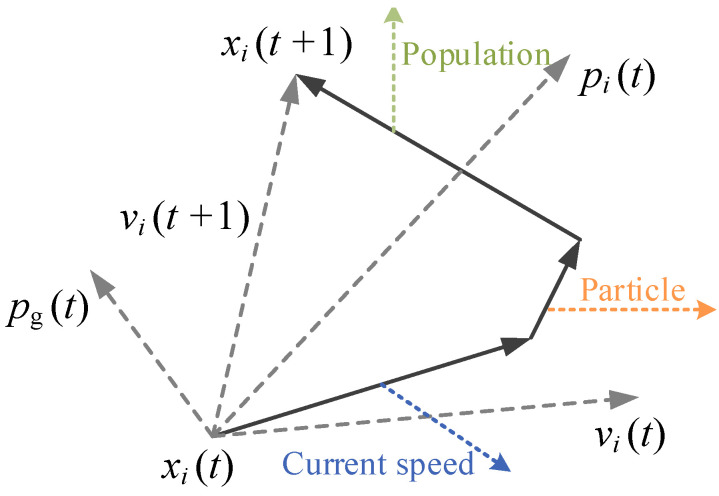
Evolutionary update diagram of particle position.

PSO functions as a heuristic intelligent optimization technique inspired by the collective intelligence observed in flocks of birds, fish, and groups of humans. During the quest for optimal solution, each particle lacks knowledge of exact location of optimal solution but is aware of the positional relationship between its current position and the optimal solution. Furthermore, the entire particle swarm can ascertain the position of particle closest to optimal solution within swarm during each iteration through collaborative mechanisms. Subsequently, while seeking the optimal solution, each particle determines its subsequent flight direction and speed based on its individual optimal position, flight direction, and those of the overall population, as depicted in Figure 2.

The stochastic particle swarm algorithm (SPSO) [24] is developed on the basis of the standard particle swarm algorithm, which changes the original straight-line search and avoids the “premature” problem of standard PSO. The PSO problem assumes that in a D-dimensional target search space, there are *N* particles forming a community, and $X_{i}=\left(x_{i1},x_{i2},\cdots x_{iD}\right)\;\;,\;\;i=1,2,\cdots ,N$ is the *i*-th particle in a D-dimensional vector. The “flying” speed of the *i*-th particle is also a D-dimensional vector, and $V_{i}=\left(v_{i1},v_{i2},\cdots ,v_{iD}\right)\;\;,\;\;i=1,2,\cdots ,N$ is the “flying” speed of the *i*-th particle in a D-dimensional vector.

Let $P_{best}=\left(P_{i1},P_{i2},\cdots ,P_{iD}\right)\;\;,\;\;i=1,2,\cdots ,N$ be the individual extreme value, which refers to the optimal position searched so far by the *i*-th particle, and $g_{best}=\left(P_{g_{1}},P_{g_{2}},\cdots ,P_{g_{D}}\right)$ is the global extreme value, which refers to the optimal position searched so far by the entire particle swarm.

When these two optimal values are found, the particle updates its speed and position according to the following formula:

(7) $$v_{id}=\omega \times v_{id-1}+c_{1}r_{1}\left(p_{id}-x_{id}\right)+c_{2}r_{2}\left(p_{gd}-x_{gd}\right)$$

(8) $$x_{id+1}=x_{id}+v_{id}\;$$

where *v* is the speed of the particle; *ω* is a weighting coefficient and its value is generally taken as a number from 0.1 to 0.9; *x* is the current position of the particle; and *r*_1_, *r*_2_ are random numbers uniformly distributed between 0 and 1. *c*_1_ and *c*_2_ are called the learning factor, usually taken as 2. In sociology, *c*_1_ and *c*_2_ represent the ability to summarize oneself and learn from outstanding individuals in the group, respectively. The right side of Equation (6) consists of three parts: “inertia” or “momentum” is the first part, which represents the tendency of particles to maintain their previous speed, reflecting a particle’s “habits” of motion; “cognition” is the second part, which represents the tendency of particles to approach their best historical position, reflecting a particle’s memory or recollection of its own historical experience; and “society” is the third part, representing the tendency of particles to approach the best historical position of a group or neighborhood, which reflects the group’s historical experience of collaboration and knowledge sharing among particles.

When *ω* = 0, the flight speed of the particle only depends on the current position of the particle, the historical best position, and the historical best position of the particle group. The speed itself has no memory. In this way, the particle located in the global best position will remain stationary, while other particles tend to go to their own best positions and the weighted center of the global best position. The new evolution equation is as follows:

(9) $$v_{i}(t+1)=c_{1}r_{1}\left(p_{i}(t)-x_{i}(t)\right)+c_{2}r_{2}\left(p_{g}(t)-x_{i}(t)\right)$$

(10) $$x_{i}(t+1)=x_{i}(t)+v_{i}(t+1)\;$$

Therefore, the new evolutionary equation weakens the global search ability and strengthens the local search ability. At the same time, when *x_i_* = *p_i_* = *p_g_*, the particles will stop evolving. In order to improve the global search capability, *p_g_* can be retained as the historical best position of the particle group, and the positions of particles are re-randomly generated in the search space.

SPSO is widely used because of its efficient search capabilities. It can find the best solutions among multiple targets and can simultaneously find multiple Pareto optimal solutions in a parallel manner, thereby improving the efficiency and accuracy of solutions. SPSO has a wide range of applications. It has the ability to handle various types of objective functions and constraints. It can be combined with traditional optimization techniques to improve efficiency and overcome limitations and plays a huge role in multi-objective optimization problems.

#### 2.2.2. KF Model

Kalman filtering technology (KF) is an efficient recursive filter (i.e., updating the current time estimation based on the previous time estimation), which can estimate the state of the dynamic system from a series of noisy observation data. Its main advantage is that it can handle noise and uncertainty, while providing the ability to update in real time. With the development of technology, Kalman filter and its variants are more and more widely used in modern engineering and science.

The dynamic system described by the following state space model is considered, including state equation and observation equation [25]. **Φ** is the state transition matrix, **Γ** is the noise driving matrix, and **H** is the observation matrix [26].

(11) $$X\left(k+1\right)=\Phi X\left(k\right)+\Gamma W\left(k\right)$$

(12) $$Z\left(k\right)=HX\left(k\right)+V\left(k\right)$$

where *k* denotes the discrete time, and **X**(*k*) and **Z**(*k*) correspond to the state vector and measurement information of the dynamic system at time *k*, respectively. **W**(*k*) and **V**(*k*) represent process noise and measurement noise, respectively. 

In the derivation of KF model, the following two assumptions need to be established [27]:

**Assumption** **1.** *The process noise **W**(k) and the measurement noise **V**(k) are uncorrelated Gaussian noises with a mean of zero and variance of **Q** and **R**, respectively.*

**Assumption** **2.** *The initial state **X**(0) of the dynamic system is not related to the process noise **W** (k) and the measurement noise **V**(k).*

The Kalman filtering problem can be expressed as follows. Based on the measurement information {**Z**(1), **Z**(2), …, **Z**(*k*)}, the linear minimum variance estimation result $\hat{X}\left(j/k\right)$ of the state vector **X**(*j*) is obtained, and the minimization index can be expressed as follows:

(13) $$J=E\left[{\left(X\left(j\right)-\hat{X}\left(j/k\right)\right)}^{T}\left(X\left(j\right)-\hat{X}\left(j/k\right)\right)\right]$$

For *j* > k, *j* = k, and *j* < k, corresponding to the predictor, filter, and smoother, respectively. The predictor mainly forecasts the future state. The filter mainly processes the state noise of the current moment to obtain the optimal estimation of the current moment in real time. The smoother makes an accurate estimate of the present moment state based mainly on future information.

Under the performance index, the problem can be reduced to finding projective as follows:

(14) $$\hat{X}\left(j/k\right)=\mathrm{proj}\left(X\left(j\right)/Z\left(1\right),Z\left(2\right),\cdot \cdot \cdot \;,Z\left(k\right)\right)$$

Under the premise of Assumptions 1 and 2, the KF algorithm can finally be expressed in the following form.

One-step prediction of state equation:

(15) $$\hat{X}\left(k+1/k\right)=\Phi \hat{X}\left(k/k\right)$$

Status update:

(16) $$\hat{X}\left(k+1/k+1\right)=\hat{X}\left(k+1/k\right)+K\left(k+1\right)\hat{Z}\left(k+1\right)$$

(17) $$\hat{Z}=Z\left(k+1\right)-H\hat{X}\left(k+1/k\right)$$

Filter gain matrix:

(18) $$K\left(k+1\right)=P\left(k+1/k\right)H^{T}s{\left(k\right)}^{-1}$$

(19) $$s\left(k\right)=HP\left(k+1/k\right)H^{T}+R$$

One step prediction of covariance matrix

(20) $$P\left(k+1/k\right)=\Phi P\left(k/k\right)\Phi^{T}+\Gamma Q\Gamma^{T}$$

Covariance matrix update:

(21) $$P\left(k+1/k+1\right)=\left[I_{n}-K\left(k+1\right)H\right]P\left(k+1/k\right)$$

In a complete filtering cycle, the calculation process of Kalman filtering algorithm can be obviously divided into two different stages: the time update stage and the measurement update stage, according to the sequence of the dynamic system information and the external measurement information.

## 3. Results and Discussion

### 3.1. Parameter Setting

**Figure 3:**
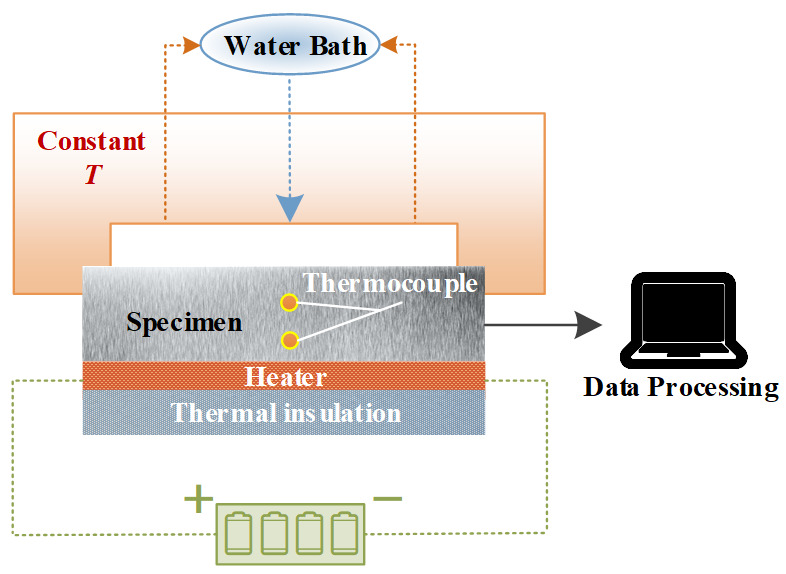
Schematic diagram of experimental system [28].

Figure 3 shows a detailed schematic diagram of the experimental device [28]. The transient heat flow is simulated by installing a thin film heater on the front surface of the specimen, and the rear surface of the specimen is continuously cooled by a constant-temperature water mist. In addition, thermocouples are installed 5 mm and 10 mm from the heating surface to obtain the measurement information.

The thickness, density, specific heat capacity, and true thermal conductivity of the medium during the reconstruction process are *L_x_* = 0.02 m, *ρ* = 3120 kg/m^−3^, *c*_p_ = 837 J/(kg∙K), and λ = 40 W/(m∙K), respectively. The initial temperature distribution of the medium and the natural convection heat transfer at the left and right boundaries are *T*_0_ = 300 K and *h*_1_ = *h*_2_ = 8 W/(m^2^∙K), respectively, and the emissivity of the left and right boundaries of the medium is 1. The number of discrete grids is N*x* = 50, the number of discrete time layers is N*t* = 5000, and the time step is d*t* = 0.1 s. The real time-varying heat flux acting on the left boundary of the medium is shown in the following formula:

(22) $$q\left(t\right)=\left\{\begin{array}{l} 140000\;\;\;\;W{/m}^{2}\;\;\;\;\;500\leq t_{\mathrm{line}}\leq 1500\\ 80000\;\;\;\;\;\;W{/m}^{2}\;\;\;\;\;\;\;\;\;\;\;\;\mathrm{other} \end{array}\right.$$

In the actual process, the existence of equipment errors and human errors is inevitable. Therefore, Gaussian white noise must be added to the temperature signal obtained by the simulation calculation so that the temperature measurement signal in actual engineering can be simulated more realistically and accurately. The temperature measurement signal with Gaussian white noise can be expressed by the following relationship:

(23) $$T_{m2}=T_{m1}+\sigma \varsigma$$

where *T*_m2_ is the measured temperature after adding the measurement noise; *T*_m1_ represents the precise value of the boundary temperature calculated by forward modeling; *ς* represents a random array consistent with a normal distribution with a mean value of 0 and a standard deviation of 1; and *σ* is the standard deviation.

The transient heat flow and internal thermal conductivity of the medium boundary are reconstructed based on the simulated measurement signals. In order to effectively evaluate the quality of the reconstruction results, an evaluation factor is introduced between the reconstruction target coefficient and the real coefficient, that is, the relative error, as shown below:

(24) $$\varepsilon =\left|\frac{x_{r}-x_{t}}{x_{t}}\right|$$

where *x_r_* and *x_t_* represent the reconstructed value and exact value of the target coefficient, respectively.

### 3.2. Reconstruction Results of Pure Heat Conduction Problem

On the basis of the simulation process of the aforementioned forward problem and the SPSO solving model above, the time-varying distribution of the internal thermal conductivity of the translucent medium and the boundary heat flux density were reconstructed and studied. In the research process, the actual values of the multi-objective parameters are given in advance. Through a forward simulation, the temperature information at the specific location of the medium is obtained, which is used as the measurement signal required for the real-time reconstruction of the boundary heat flow and thermal properties.

**Figure 4:**
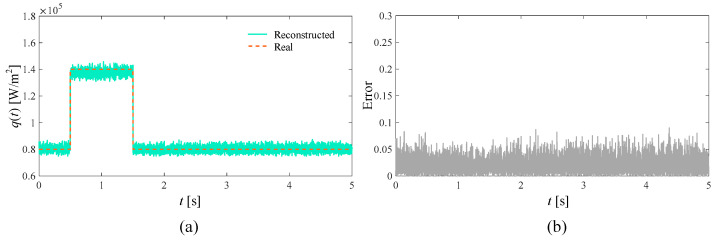
The ideal reconstruction result. (**a**) is the comparison between the reconstructed boundary heat flow and the real heat flow; (**b**) is the relative error of the reconstructed results.

**Figure 5:**
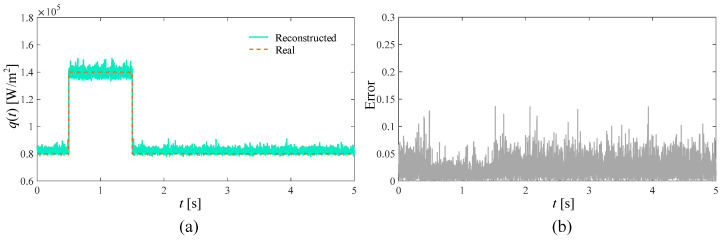
Reconstruction results with 3% noise added. (**a**) is the comparison between the boundary heat flow reconstructed after adding noise and the real heat flow; (**b**) is the relative error of the results.

**Figure 6:**
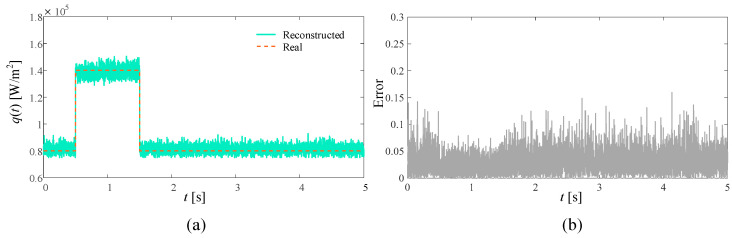
Reconstruction results with 5% noise added. (**a**) is the comparison between the boundary heat flow reconstructed after adding noise and the real heat flow; (**b**) is the relative error of the results.

**Figure 7:**
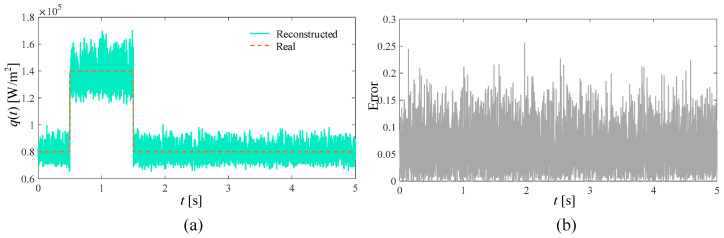
Reconstruction results with 10% noise added. (**a**) is the comparison between the boundary heat flow reconstructed after adding noise and the real heat flow; (**b**) is the relative error of the results.

**Figure 8:**
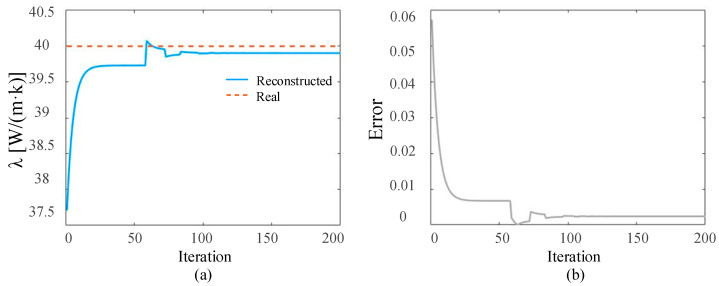
Under ideal conditions, the reconstructed results in the iterative process. (**a**) is the comparison between the reconstructed thermal conductivity and the real thermal conductivity; and (**b**) is the relative errors of the reconstructed results.

**Figure 9:**
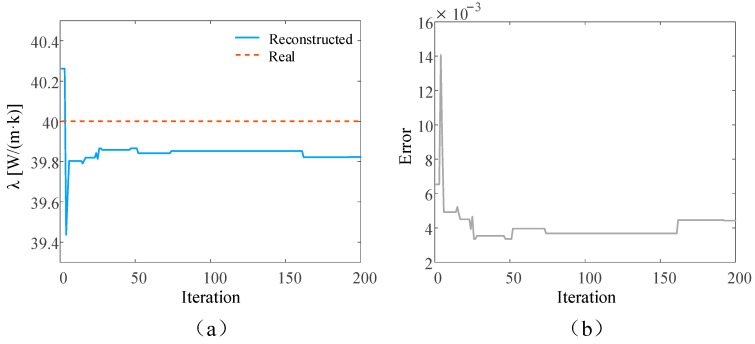
Reconstruction results with 3% noise added. (**a**) is the comparison between the reconstructed thermal conductivity and the real thermal conductivity after adding noise; (**b**) is the relative error of the results.

**Figure 10:**
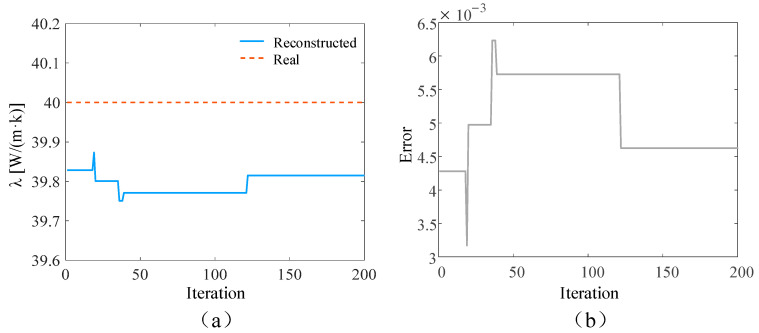
Reconstruction results with 5% noise added. (**a**) is the comparison between the reconstructed thermal conductivity and the real thermal conductivity after adding noise; (**b**) is the relative error of the results.

**Figure 11:**
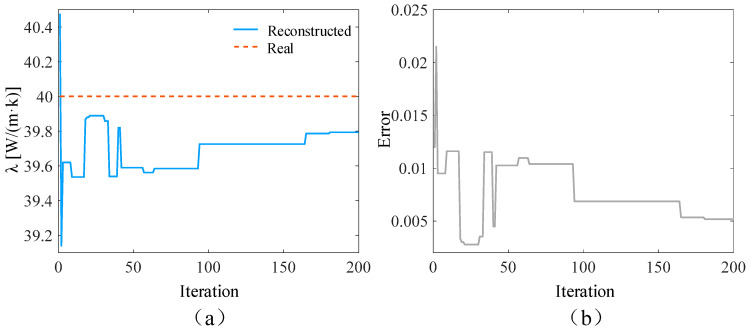
Reconstruction results with 10% noise added. (**a**) is the comparison between the reconstructed thermal conductivity and the real thermal conductivity after adding noise; (**b**) is the relative error of the results.

In the ideal situation without adding noise and in the presence of 3%, 5%, and 10% measurement noise, respectively, the SPSO algorithm is used to simultaneously reconstruct the time-varying heat flow *q*(*t*) at the medium boundary and the internal thermal conductivity λ. The number of populations in the inversion model is set to 15, the self-learning factor is two, and the group learning factor is two. The reconstructed boundary heat flow and relative error when the number of layers is 5000 under different circumstances are shown in Figure 4, Figure 5, Figure 6 and Figure 7, respectively. Additionally, the reconstructed thermal conductivity and relative error when the number of iterations is 200 are shown in Figure 8, Figure 9, Figure 10 and Figure 11, respectively.

From Figure 4, Figure 5, Figure 6 and Figure 7, with the increase in random measurement noise, the oscillation effect of the boundary heat flow near the actual value in the convergence process deteriorates significantly. At the same time, the relative error margin also increased, and the maximum error value increased from about 0.05 to about 0.25. But, fortunately, even with the addition of 10% measurement noise, more reasonable heat flow reconstruction results can still be obtained based on the current algorithm, indicating that the tracking ability of the established inversion model can effectively deal with the impact of increased random measurement noise.

As can be seen from the reconstructed results of thermal conductivity in Figure 8, Figure 9, Figure 10 and Figure 11, with the increase in random measurement noise, the deviation degree of the reconstructed thermal conductivity from the real value increases slightly, and the reconstructed value of thermal conductivity changes from 39.90 W/(m∙K) to 39.79 W/(m∙K). On the other hand, the convergence value of the relative error also increases slightly with the addition of measurement noise, and the maximum error value increases from about 0.003 to 0.005. Even when 10% measurement noise is added, the thermal conductivity can still be accurately reconstructed based on the current algorithm, indicating that the established inversion model’s ability to track the thermal conductivity is relatively weakly affected by random measurement noise.

Table 1 reflects the model reconstruction time after adding different measurement errors; the objective function value when the inversion calculation reaches convergence; the relative error value of the thermal conductivity coefficient; and the average relative error value of the boundary time-varying heat flow at this time. Since the discrete time layer selected for the model is larger, the overall calculation time is longer. The convergence value (optimal fitness) of the objective function decreases as the measurement error increases.

On the whole, when the established SPSO model is used to simultaneously reconstruct the internal thermal conductivity of the model and the boundary time-varying heat flow, the inversion accuracy of the thermal conductivity is significantly better than the inversion ability of the boundary heat flow. Taken together, when the established PSO model is used to simultaneously reconstruct the internal thermal conductivity of the model and the boundary time-varying heat flow, the inversion accuracy of the thermal conductivity is significantly better than the inversion ability of the boundary heat flow. Even if 10% measurement noise is introduced into the model, the relative error of the thermal conductivity is 0.53%, and the average relative error of the boundary time-varying heat flow is 5.16%. The reconstruction accuracy of different target parameters is still within the ideal range, which proves that the established model, which simultaneously reconstructs multiple quantities of thermal radiation, has good levels of robustness and effectiveness.

### 3.3. Reconstruction Results of Radiation Thermal Coupling Problem

**Figure 12:**
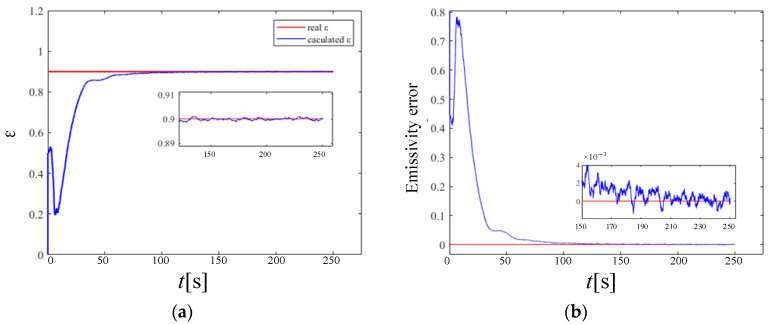
Emissivity results from reconstruction of multiple parameters. (**a**) is the iterative convergence process of emissivity; (**b**) is the reconstruction error of emissivity.

**Figure 13:**
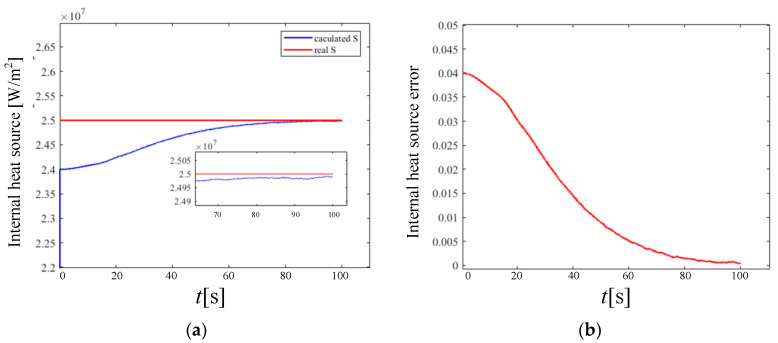
The internal heat source results from the reconstruction of multiple parameters. (**a**) is the iterative convergence process of the internal heat source; (**b**) is the reconstruction error of the internal heat source.

Under a high temperature, the heat transfer of a porous material solid wall is composed of two parts: radiation and heat conduction. At this time, the separate heat conduction problem can no longer meet the actual needs of industry. Therefore, this section performs a simultaneous inversion of the semi-transparent solid wall emissivity and internal heat source based on the radiation-to-thermal coupling model and the KF model. The reconstruction time is 500 s. The final reconstruction results and errors of thermal radiation characteristics are shown in Figure 12 and Figure 13.

In the KF model of the multi-volume reconstruction at the same time, compared with the emissivity, the convergence speed of the reconstruction of the heat source in the medium is relatively slow, but the convergence process is smoother. When the calculation reaches convergence, the reconstruction error of the wall emissivity and the internal heat source is in the order of 10^−3^, which proves the accuracy of the established multi-volume model of thermal radiation characteristics.

**Figure 14:**
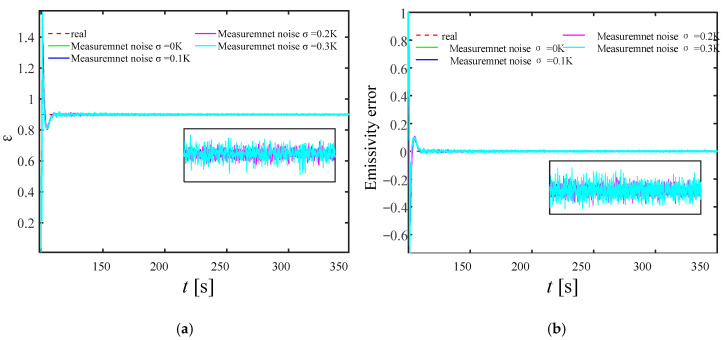
Emissivity reconstruction results with different measurement noise. (**a**) is the iterative convergence process of emissivity; (**b**) is the reconstruction error of emissivity.

**Figure 15:**
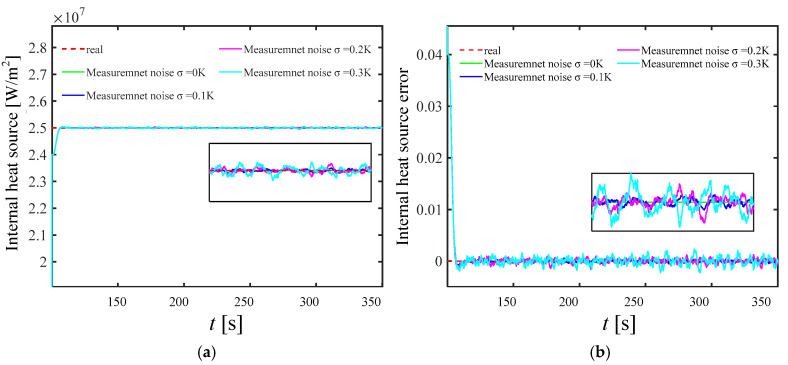
Reconstruction results of internal heat source with different measurement noise. (**a**) is the iterative convergence process of the internal heat source; (**b**) is the reconstruction error of the internal heat source.

In engineering practice, measurement noise is often unavoidable. Therefore, in order to verify the effectiveness of the current KF reconstruction model, it is necessary to analyze the influence of measurement noise on the reconstructed emissivity and internal heat source distribution in detail. Figure 14 and Figure 15 show the wall emissivity and internal heat source distribution and calculation error based on the KF reconstruction under different measurement noises of 0.1 K, 0.2 K, and 0.3 K, respectively. It can be found from the figure that even if measurement errors are added, the reconstructed internal heat source and emissivity values gradually converge to the real value within 150 s after the start of reconstruction, which proves that the model has a certain near-real-time reconstruction ability and a good level of robustness.

As the level of measurement noise rises, the algorithm’s stability is somewhat impacted, leading to fluctuations in the reconstruction results around the actual value. However, the reconstruction error remains within 1%, indicating it has a satisfactory level of credibility and maintains an ideal level of accuracy. On the other hand, the time delay of the reconstruction parameters almost does not change with the measurement error. This is because in the current study, it is assumed that the measurement noise and its covariance matrix are independent of each other, and the Kalman filter gain is only related to the covariance of the measurement noise and has nothing to do with itself, so the measurement noise change does not affect the tracking ability of the KF technology.

**Figure 16:**
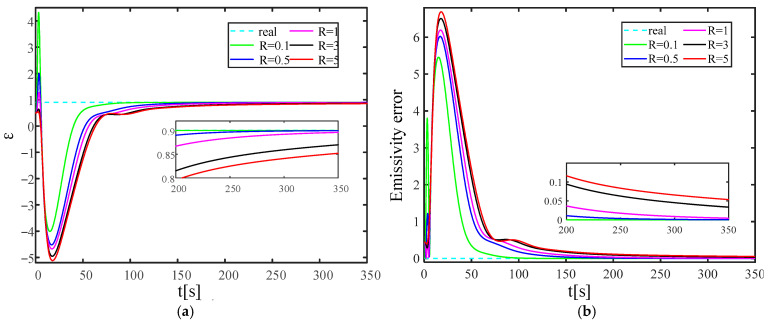
Emissivity reconstruction results with different levels of measurement error covariance. (**a**) is the iterative convergence process of emissivity; (**b**) is the reconstruction error of emissivity.

**Figure 17:**
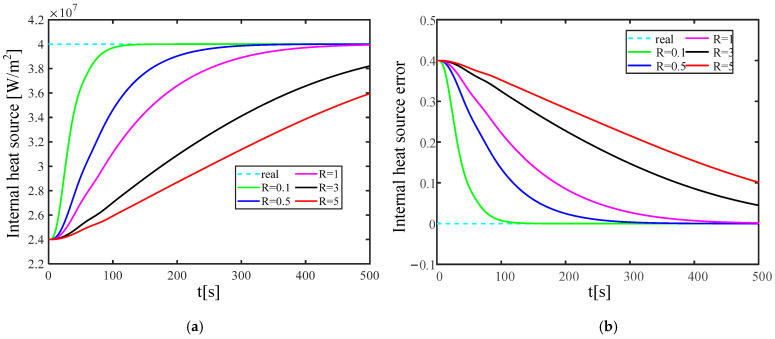
Reconstruction results of internal heat source under different levels of measurement error covariance. (**a**) is the iterative convergence process of the internal heat source; (**b**) is the reconstruction error of the internal heat source.

The KF gain has a strong correlation with the measurement noise covariance. It is necessary to study the influence of the measurement noise covariance change on the reconstruction quality and speed of the established solid wall radiation characteristic parameter inversion model. Figure 16 and Figure 17 show the wall emissivity and internal heat source reconstruction process and calculation error based on Kalman filter technology when the measurement error covariance values are 0.1, 0.5, 1, 3, and 5, respectively.

The calculation results demonstrate that as the measurement error covariance increases, the convergence speed towards the actual characteristic parameters progressively decelerates. The process of reconstructing the emissivity can approach the real emissivity more smoothly within 250 s. When the measurement error covariance R = 5, the error of the emissivity reconstruction convergence is near 5%, which is within the ideal error range. Compared with the emissivity, after adding the same measurement error covariance, the reconstruction quality of the internal heat source is relatively low, and the reconstruction convergence speed is relatively slow. When R ≤ 1, the process of reconstructing the internal heat source can smoothly approach the real internal heat source within 500 s, and the reconstruction error does not exceed 5%. When R > 1, there is still a gap between the process of reconstructing the internal heat source and the true value within 500 s. Currently, the reconstruction error is about 10%, which is still within the acceptable range.

## 4. Conclusions

This work focuses on the multi-parameter reconstruction of time-varying photothermal properties in participating media. Initially, a multi-parameter reconstruction model for the pure thermal conductivity issue is developed utilizing SPSO. Subsequently, multi-objective inversion optimization is achieved through the application of KF in addressing the coupling problem of radiation thermal conductivity. The results indicate that the inversion effect of the thermal conductivity is better than that of the boundary time-varying heat flux. As the measurement noise increases, the reconstruction accuracy and convergence stability of the thermal conductivity and the boundary time-varying heat flow with a discrete dimension of 5000 are reduced to varying degrees. In particular, the reconstruction effect of the boundary time-varying heat flow has a stronger oscillation or even divergence. After introducing 10% measurement noise, the relative error data of different target parameters are still in the ideal range, which proves the robust effect of the time-varying multi-variable model reconstructed by SPSO. 

In addition, the results of the simultaneous reconstruction of multiple total thermal radiation characteristics of solid wall objects based on KF technology are close to the real values. The model has a high level of reliability as the reconstruction error is close to zero. Even if the internal heat source has a crosstalk effect on the reconstruction of the surface emissivity, this does not significantly affect the reconstruction accuracy of the internal heat source. With the increase in measurement noise, the reconstruction process still has a high level of computational efficiency, which makes near-real-time reconstruction possible. Moreover, the change in measurement noise does not affect the tracking ability of the inversion algorithm. The re-construction convergence stage only generates more obvious fluctuations with the increase in noise, but the reconstruction error is still within the ideal range, and the reconstruction result is reliable, which proves the robustness of the model.

## Figures and Tables

**Table 1:** Related reconstruction data under different measurement errors.

Measurement Error	Time (s)	Object Function	Error (λ)	Average Error (*q*)
0%	6074.2	6.1472 × 10^−26^	0.0036	0.0392
3%	6138.9	8.7431 × 10^−20^	0.0043	0.0447
5%	6346.2	6.5001 × 10^−16^	0.0046	0.0494
10%	6675.5	3.2831 × 10^−13^	0.0053	0.0516

## Data Availability

Data are contained within the article.
